# General, spinal or regional anaesthesia does not affect strength performance 6 months after ACL reconstruction

**DOI:** 10.1007/s00167-022-07052-w

**Published:** 2022-07-30

**Authors:** M. Wenning, M. Mauch, A. H. Heitner, S. Heinrich, G. N. Sofack, M. Behrens, R. Ritzmann

**Affiliations:** 1 Rennbahnklinik, Muttenz, Basel, Switzerland; 2 Department of Orthopedic and Trauma Surgery, Faculty of Medicine, University Medical Center, University of Freiburg, Hugstetter Str. 55, 79104 Freiburg, Germany; 3 Department of Anesthesiology and Intensive Care Medicine, Faculty of Medicine, University Medical Center, University of Freiburg, Freiburg, Germany; 4 Institute of Medical Biometry and Statistics, Faculty of Medicine, University of Freiburg, Freiburg, Germany; 5 Department of Biomechanics, Rennbahnklinik, Kriegackerstrasse 100, Muttenz, CH-4132, Basel, Switzerland; 6 Department of Sport and Sport Science, University of Freiburg, Freiburg, Germany

**Keywords:** Perioperative anaesthesia, ACL reconstruction, Isokinetic strength, Return-to-sports

## Abstract

**Purpose:**

The recovery of strength is a key element in successfully returning to sports after ACL reconstruction. The type of anaesthesia has been suspected an influential factor in the post-operative recovery of muscle function.

**Methods:**

In this retrospective analysis, *n* = 442 consecutive patients undergoing primary isolated ACL reconstruction using a hamstring autograft were analysed by pre- and post-operative isokinetic tests in a single orthopaedic centre. These were subdivided into four cohorts: (1) general anaesthesia (*n* = 47), (2) general anaesthesia with prolonged (48 h) on-demand femoral nerve block (*n* = 37), (3) spinal anaesthesia (*n* = 169) and (4) spinal anaesthesia with prolonged (48 h) on-demand femoral nerve block (*n* = 185). Primary outcome was the change from pre- to post-operative isokinetic strength during knee extension and flexion.

**Results:**

Using one-way ANOVA, there was no significant influence of the type of anaesthesia. The main effect of anaesthesia on change in extension forces was not significant, and effect sizes were very small (n.s.). Similarly, the main effect of anaesthesia on change in flexion forces was statistically not significant (n.s.).

**Conclusions:**

The findings of this study support the interpretation that the type of anaesthesia has no significant effect on the ability to recover thigh muscle strength 6 months after isolated hamstring ACL reconstruction. With regard to the recovery of athletic performance and return-to-sports testing criteria, there is no reason to avoid regional anaesthesia.

**Level of evidence:**

III.

## Introduction

Rupture of the anterior cruciate ligament (ACL) is one of the most frequent ligamentous injuries in sports, which is treated by arthroscopic ACL reconstruction under anaesthesia in the vast majority of patients [14]. Further on, rehabilitation following ACL reconstruction is guided by performance measures leading to a time- and criterion-based return to athletic activity [5, 6]. Generally, a strength deficit on the injured side compared to the healthy side of less than 15–20% is recommended before returning to running [5] and less than 10–15% (= a limb symmetry index [LSI] > 85–90%) before returning to competitive or pivoting sports [27].

Current research focuses on factors that may have an influence on return to activity testing and patient outcome [3, 5]. There has been ongoing debate whether the type of anaesthesia, regional vs. general, influences post-operative neuromuscular activation and performance [4, 9, 19, 24].

Current evidence suggests that peripheral nerve blockage may result in quadriceps weakness during the early stages of rehabilitation and may even increase the risk for an early re-rupture [4, 9, 24]. Peripheral blockage of the femoral nerve, the obturator nerve and the sciatic have been evaluated, and regional anaesthesia may result in improved pain control [18]. However, the anatomical proximity, e.g. of the obturator nerve to the vastus medialis muscle carries the risk of additional neuromechanical dysfunction of the vastus medialis including post-operative muscular atrophy and inhibition [10, 13]. Despite its benefits for pain control, post-operative mobility and its low complication rate, there is ongoing debate about the long-term influence of regional anaesthesia with regard to the recovery of athletic performance, return to activity and possible re-injury risk [9, 13, 14]. Moreover, H/Q-ratio as an indicator of ipsilateral strength imbalance may also be affected by regional procedures since, e.g. femoral nerve blockage primarily targets knee extensor muscles. Some of the studies available have included different types of grafts or not reported on graft sites at all, which may have resulted in a heterogeneous study cohort and effectively could have had an influence on post-operative strength performance [4, 9, 13, 20]. Additionally, there is evidence that preoperative strength has a significant influence on the post-operative recovery of performance, which has rarely been taken into account [8, 22]. Thus, it needs to be analysed whether the recovery of strength following ACL reconstruction is significantly altered by the choice perioperative anaesthesia.

With reference to the aforementioned most recent scientific evidence [4, 9, 13, 18, 24], the aim of this study was to evaluate the effect of spinal, regional and general anaesthesia on strength performance six months following ACL reconstruction in a homogenous and large cohort of athletic patients. For that purpose, we analysed the differences in the recovery of strength following hamstring ACL reconstruction with respect to the preoperative values. As it is known that the healthy leg improves in strength during rehabilitation also, this requires to control for potential effects between the non-affected and the injured legs. This will allow to evaluate whether spinal and/or prolonged regional anaesthesia could have a negative impact on the ability to recover and increase muscular strength during ACL rehabilitation. According to the literature, it was hypothesized that regional anaesthesia including femoral nerve blockage and prolonged on-demand peri-nerval anaesthesia will reduce knee extension and flexion strength when testing for RTP around six months post-operatively [4, 9, 13, 24].

## Materials and methods

This retrospective analysis was approved by the institution’s ethical committee (EKNZ 2017–01,825), and all participants had given written general consent to participate. The study is registered in the German Clinical Trial Register (DRKS 00020210) and was performed according to the Declaration of Helsinki.

### Study population

A continuous cohort of *n* = 442 continuous patients suffering a primary traumatic ACL rupture was analysed. All patients had undergone functional testing pre- and post-operatively. Arthroscopic ACL reconstruction was performed in a sports orthopaedic centre by experienced surgeons from 11/2017 to 12/2020.

The inclusion criteria were primary ACL reconstruction using ipsilateral hamstring autograft with no associated lesions that would have been relevant to post-operative rehabilitation. Accordingly, exclusion criteria were: (a) any associated lesion requiring additional surgical intervention with effect on the post-operative mobilization or weight bearing like meniscal repair, high tibial osteotomy, cartilage interventions such as microfracturing, larsons’ repair or multiligament trauma [4, 25], (b) revision cases, and (c) missing data.

Baseline preoperative characteristics of patients, namely age, sex and body mass index, were compared between the four groups, and no statistically significant differences were found. Hence, no further matching procedures were performed.

### Anaesthesiological proceeding

The population was divided into four groups (see Table 1) according to the type of anaesthesia received during and after the surgical reconstruction as follows:

1. general anaesthesia without regional anaesthesia (GA),
2. general anaesthesia with a prolonged peripheral femoral nerve block over 48 h on demand using a peri-nerval catheter (GA + *pFNB),*
3. spinal anaesthesia without additional regional anaesthesia (Spinal).
4. spinal anaesthesia with a prolonged peripheral femoral nerve block over 48 h on demand using a peri-nerval catheter (Spinal + *pFNB).*

**Table 1:** Overview of the composition of the subgroups

Variable	GA *n* = 47		GA + pFNB *n* = 37		Spinal *n* = 169		Spinal + pFNB *n* = 185	
Sex	*n*	*%*	*n*	%	*n*	%	*n*	%
Male	35	74	27	73	129	76	124	67
Female	16	34	10	27	40	24	61	33
Age at surgery (in y, Median (IQR))								
10–19	16	34	8	22	22	13	30	16
20–29	18	38	16	43	83	49	70	38
30–39	6	13	6	16	38	22	46	25
40–49	9	19	6	16	17	10	31	17
50–59	2	5	1	3	9	5	7	4
Height (in m, Mean (SD))	1.76 (0.08)		1.75 (0.09)		1.76 (0.09)		1.74 (0.08)	
Weight (in kg, Mean (SD))	75.2 (12.6)		77.5 (11.9)		76.9 (12.6)		75.3 (13.7)	
BMI (Median (IQR))	24.1 (3.9)		24.7 (4.7)		24.5 (4.0)		24.2 (4.3)	
Dominant leg injured	32	68	19	51	93	55	106	57

*GA* general anaesthesia, *GA + pFNB* general anaesthesia with prolonged on-demand femoral nerve block, *Sp* spinal anaesthesia, Sp + *pFNB* spinal anaesthesia with prolonged on-demand femoral nerve block

The choice of the type of anaesthesia was left to the patients’ discretion after informed consent, except when there were contraindications against either type of anaesthesia.

Femoral nerve block was performed using ultrasound-guided placement of a peri-nerval catheter without dual guidance or any other type of nerval stimulation. The preoperative single injection comprised 20 ml of 0.5% ropivacaine, following 48 h of on-demand application of ropivacaine 0.5% or 0.75% at patients’ control via a pulsatile syringe driver. The catheter was left in place for 48 h; only in the rare case of local irritation the catheter was removed earlier.

Spinal anaesthesia was performed using 0.5% carbostesin with a general dose of 12–17 mg (2.5 ml) for isolated ACL reconstruction with an estimated time of surgery below 1.5 h. At the time of choosing the type of anaesthesia, neither the patient nor the anaesthesiologist was aware of this retrospective analysis, precluding a potential influence. The type of anaesthesia was independent from the time of hospitalization (avg. 4 days) or post-operative rehabilitation protocol.

No cases of persisting neurological impairments (sensory or motor) were observed in the study cohort during the study period from pre-surgical medical assessment to 6 months after surgery.

### Rehabilitation protocol

Standardized post-surgical treatment was achieved by a homogenous controlled time- and evidence-based rehabilitation protocol. It was identical for all patients and executed with both the injured and not injured leg as previously described [22, 25, 26]. The criterion-based rehabilitation scheme of the present study contained a clustering into four phases with a progressive therapy algorithm respecting the vulnerability of the bony and ligamentous tissue. Exercises ranged from monoarticular passive treatment to multiarticular active strengthening up to functional tasks with high load [22, 26]. For example, full weight bearing was allowed as soon as clinical signs of effusion had disappeared, generally after 1–2 weeks. Patients were required to perform balance squat and y-balance test with adequate qualitative and quantitative performance, while an additional balance front hop must have been performed with stable lower leg alignment, before being allowed to return to running. Generally, this was achieved after three months post-surgery. Before recommending patients to return-to-sport-specific tasks around 6 months post-surgery, the testing required sufficient quantitative symmetry in different hop tests (balance front and side hop, modified balance side hop) and qualitatively adequate 90 square hop. At this point, the isokinetic testing was performed, also.

Patients and therapists followed detailed written information about the procedure, exercises and progression criteria by the surgeon. For each of the included participants, rehabilitation protocols were checked for consistency and compliance of the patient was controlled by the physiotherapist.

### Isokinetic strength measurement

Knee extensor and flexor strength were assessed using an isokinetic dynamometer (Humac^®^/NormTM Testing & Rehabilitation System, Computer Sports Medicine, Inc. (CSMi, Stoughton, Massachusetts, USA) according to Li and Wu [16]. The dynamometer was calibrated prior to testing sessions.

Each subject was placed in an upright sitting position, the trunk at 100° leaning against the back rest of the testing table, fixed by straps across the chest and a horizontal pad over the distal third of the thighs. The knee joint axis was aligned with the mechanical axis of the dynamometer. The shin pad was placed just superior to the medial malleolus.

Prior to each test sequence, subjects performed a standardized 10-min warm-up on a cycling ergometer (50 W) followed by three submaximal repetitions to familiarize with the testing procedure. For data assessment, we use concentric–concentric contractions at a 60°/s angular speed, in the full individual range of motion (ROM) [11]. Two sets of three repetitions with maximum effort were executed. Each trial was initiated with the unaffected limb. Between sets, patients had rest for at least 1 min.

Outcome parameters were: maximal knee extension and flexion torque normalized to body mass [Nm/kg], the H/Q-ratio [%] (Hamstrings torque/ quadriceps torque *100) and the limb symmetry index (LSI) [%] (affected limb/unaffected limb*100) for the knee extensors and flexors. For data assessment, the better of the two sets was selected.

### Statistical analysis

All statistical analyses were run as complete case analyses. The primary independent variable was the type of anaesthesia administered. The two outcomes were the maximal knee extension and flexion torque normalized to body mass ([Nm/kg]. The outcomes were calculated as the gain of strength, expressed by the torque values (flexion or extension) following the operation compared to the preoperative values. The post-operative strength alone would not have been sufficient, since the post-operative gain in strength depends on the preoperative values [15, 22]. The effect of anaesthesia on the outcomes was estimated using a one-way analysis of variance (ANOVA). All assumptions for ANOVA were tested and fulfilled. The presence of normal distributions and the number of outliers in outcomes were checked using data exploration techniques. The normality of the residuals was confirmed using the Shapiro–Wilk test, and homogeneity of variance was present as the Levene’s test suggested. Statistical analysis was conducted using R (R v. 4.1.2, [21]). Graphical display was performed using Veusz (Veusz v. 3.0.1 by Jeremy Sanders). G*Power software version 3.1.94 was used to estimate required sample size for this study. The study power was set at 95% and alpha value set at 0.05. Based on these, a minimum total sample of 280 subjects was required. Effect sizes were labelled following Field's (2013) recommendations.

## Results

A total of *n* = 442 patients were included in the analysis. *N* = 47 (11%) received general anaesthesia, *n* = 37 (8%) received GA + *pFNB*, *n* = 169 (38%) received spinal and *n* = 185 (42%) received spinal + *pFNB*. The descriptive parameters of the study population are shown in Table 1.

The torque values when measured at 6 months post-operatively showed a persisting strength deficit on the injured side; however, this deficit did not result in a significant difference between the groups. Results are displayed in Tables 2 and 3.

**Table 2:** Preoperative torque values across the subgroups

		Extension	LSI extension	Flexion	LSI flexion	H/Q ratio
		Mean ± SD (Nm/kg*100)	Mean ± SD (%)	Mean ± SD (Nm/kg*100))	Mean ± SD (%)	Mean ± SD
GA	OP	141 ± 59	73 ± 26	102 ± 41	83 ± 27	75 ± 15
*n* = 47	NOP	192 ± 48		124 ± 37		65 ± 10
GA + pFNB	OP	140 ± 51	77 ± 25	93.6 ± 35	79 ± 25	69 ± 19
*n* = 37	NOP	185 ± 46		120 ± 34		66 ± 12
Spinal	OP	150 ± 52	77 ± 20	107 ± 35	83 ± 22	74 ± 19
*n* = 169	NOP	197 ± 46		130 ± 31		67 ± 11
Spinal + pFNB	OP	131 ± 57	72 ± 26	94.6 ± 36	79 ± 25	82 ± 14
*n* = 185	NOP	180 ± 43		121 ± 27		68 ± 12

*GA* general anaesthesia, *Spinal* spinal anaesthesia, *+ pFNB* prolonged femoral nerve blockage using an on-demand femoral nerve block, *OP* operated, *NOP* not affected leg, *Nm* Newtonmeter, *SD* standard deviation, *LSI* Limb symmetry index

**Table 3:** Post-operative torque values across the subgroups

		Extension	LSI extension	Flexion	LSI flexion	H/Q ratio
		Mean ± SD (Nm/kg*100)	Mean ± SD (%)	Mean ± SD (Nm/kg*100)	Mean ± SD (%)	Mean ± SD
GA	OP	162 ± 49	80 ± 18	116 ± 31	86 ± 15	75 ± 19
*n* = 47	NOP	203 ± 46		136 ± 30		68 ± 11
GA + pFNB	OP	169 ± 50	82 ± 17	115 ± 33	84 ± 15	70 ± 16
*n* = 37	NOP	206 ± 47		136 ± 32		67 ± 12
Spinal	OP	164 ± 50	80 ± 18	122 ± 31	88 ± 14	78 ± 20
*n* = 169	NOP	206 ± 47		139 ± 39		68 ± 10
Spinal + pFNB	OP	156 ± 50	81 ± 19	115 ± 27	87 ± 13	79 ± 25
*n* = 185	NOP	194 ± 41		133 ± 26		70 ± 11

*GA* general anaesthesia, *Spinal* spinal anaesthesia, *+ pFNB* prolonged femoral nerve blockage using an on-demand femoral nerve block, *OP* operated, *NOP* not affected leg, *Nm* Newtonmeter, *SD* standard deviation, *LSI* Limb symmetry index

**Fig. 1:**
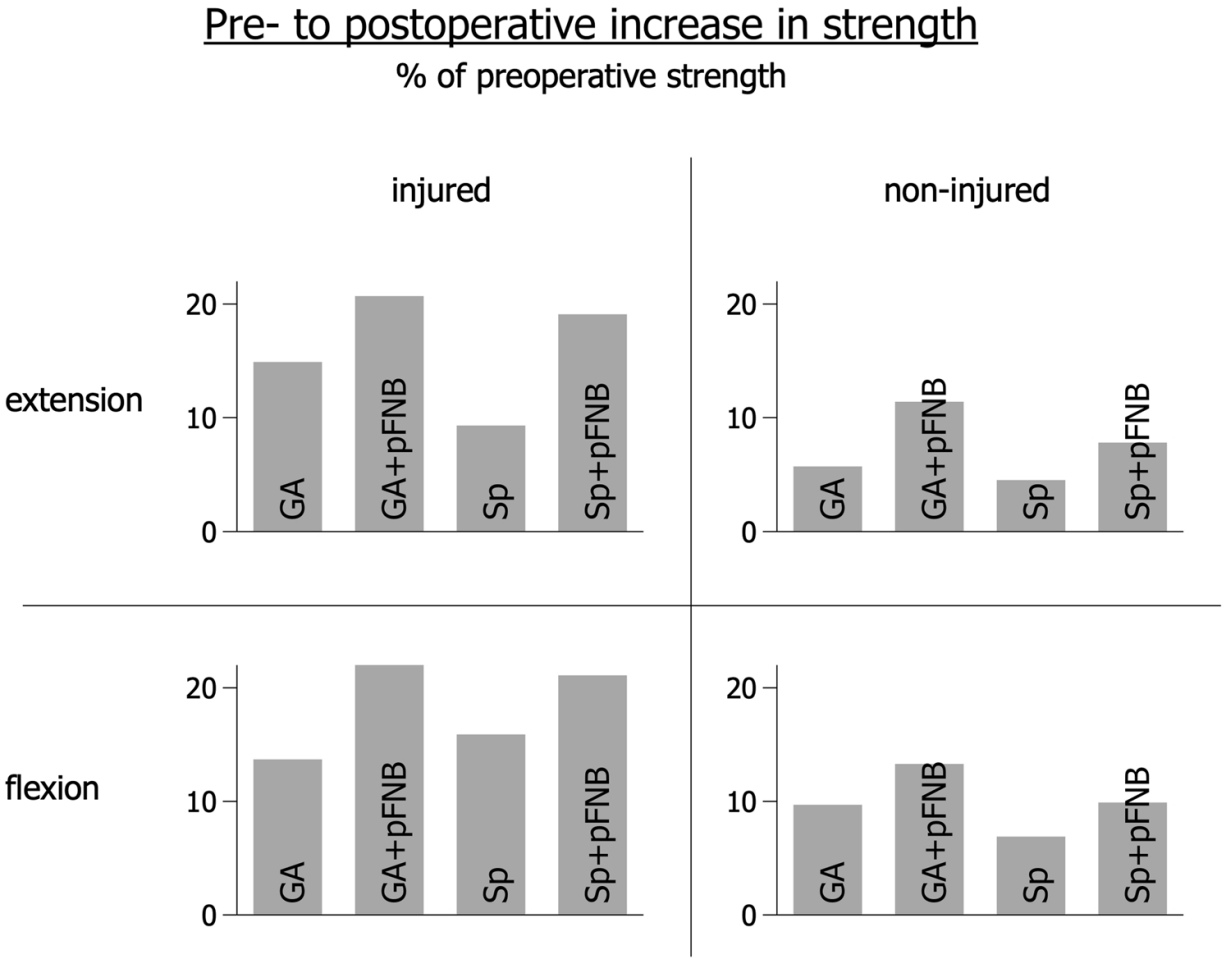
Relative increase in knee extension and flexion strength across the groups subdivided between injured and non-injured legs (% of the preoperative values). *GA* general anaesthesia, *GA + pFNB* general anaesthesia with prolonged on-demand femoral nerve block, *Sp* spinal anaesthesia, *Sp + pFNB* spinal anaesthesia with prolonged on-demand femoral nerve block

**Fig. 2:**
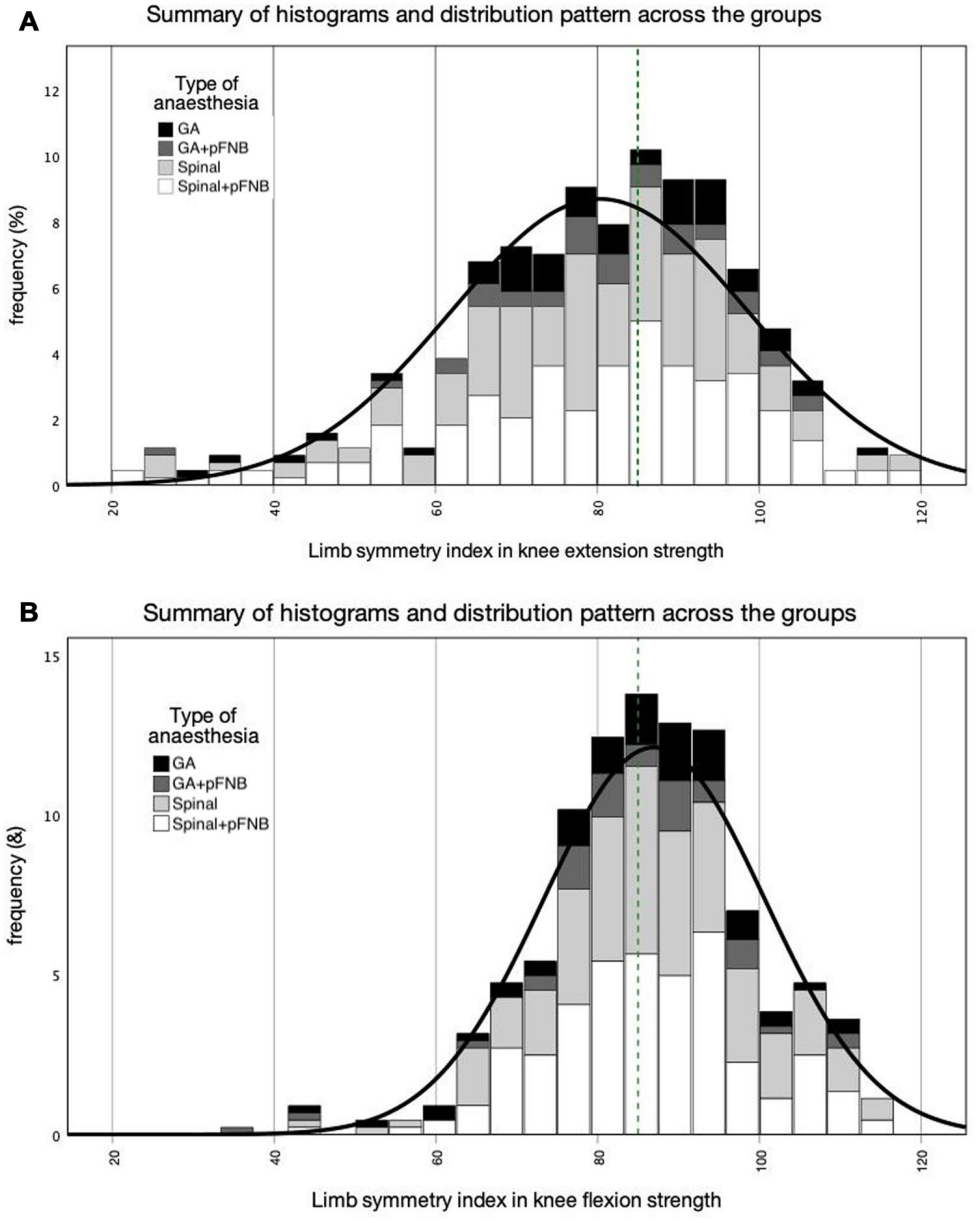
**a** Distribution of LSI in knee extension strength across the groups. dashed line = 85% LSI, black line = summary of distribution pattern, stacks = % of cases (frequency) in each group according to LSI. *GA* general anaesthesia, *GA + pFNB* general anaesthesia with prolonged on-demand femoral nerve block, *Spinal* spinal anaesthesia, *Spinal + pFNB* spinal anaesthesia with prolonged on-demand femoral nerve block. **b** Distribution of LSI in knee flexion strength across the groups. Dashed line = 85% LSI, black line = summary of distribution pattern, stacks = % of cases (frequency) in each group according to LSI. *GA* general anaesthesia, *GA + pFNB* general anaesthesia with prolonged on-demand femoral nerve block, *Spinal* spinal anaesthesia, *Spinal + pFNB* spinal anaesthesia with prolonged on-demand femoral nerve block

The main effect of anaesthesia on the change in knee extension torques was statistically not significant with very small effect sizes (n.s.). Similarly, the main effect of anaesthesia on the change in knee flexion torques was statistically not significant with again very small effect sizes (n.s.). The relative increase in strength was generally higher in the groups using prolonged post-operative anaesthesia; however, these differences were not significant (n.s.) between the groups. Figure 1 summarizes these results and displays the relative increase in strength (compared to the preoperative values) for each group and outcome variable. Figure 2a, b shows the histograms (frequency-%) of LSI across all groups in knee extension strength (a) and knee flexion strength (b).

## Discussion

The main finding of this study is that the choice of anaesthesia has no singular significant effect on the knee extension or flexion strength at 6 months post-operatively. In clinical practice, this empowers patients and treating physicians to choose the best type or combination of anaesthesia with no adverse effect on the muscular strength when returning to play.

With respect to the existing literature, this finding is surprising since it has been discussed recently that the use of peripheral nerve blockage might increase post-operative muscular atrophy and possibly the risk of re-injury [4, 9]. Therefore, it was debated whether regional anaesthesia should be performed in those athletes requiring a fast recovery in lower leg strength [3, 20]. The previous studies suffered from deficits such as heterogeneous or small cohorts, differing follow-up times or differences in types of anaesthesia [1, 4, 23]. Furthermore, they did not reflect upon the preoperative performance level. The present study was realized in a relatively large sample of well-defined, homogenous cohorts of isolated primary ACL ruptures, which were following the same rehabilitation scheme. Additionally, this analysis is the first of its type that included the preoperative strength as a reference measure, which further increases the strength of the findings. The choice of the relative increase in % of preoperative strength was defined as the most suitable primary outcome, since it values the preoperative strength in the individual’s potential to increase their strength following the different types of anaesthesia. Since it has been shown that both legs, healthy and injured, increase their strength during the rehabilitation, the isolated focus on post-operative limb symmetry or strength would have fallen short. The present study also offers an additional insight, since spinal anaesthesia and general anaesthesia were each applied singularly and also combined with regional procedures.

In order to account for intra-individual imbalances, the ipsilateral H/Q-ratio was calculated, which neither displays a significant difference between the groups post-operatively. If femoral nerve blockage had a significant effect on post-operative strength, it was hypothesized that extensor strength would be primarily affected and therefore induce a side-to-side difference in post-operative H/Q-ratio, which was not the case in this study.

Previous studies have mainly focused on the early post-operative performance; some of them with regard to time intervals of 1 to 6 weeks post-operatively [4]. This time span, however, is too early for decision-making in return-to-sports testing. Magnussen et al. found a reduction in strength at 6 weeks due to femoral nerve blocks, which had resolved by 6 months post-operatively [17].

If the peripheral anaesthetic agents had a negative effect on strength, we would have expected that the group solely receiving general anaesthesia without any perinerval injection would have been superior, which is certainly not the case. Moreover, the application of a prolonged femoral nerve block (FNB) should have affected knee extensions torques primarily, but the ability to regain or increase strength was comparable between knee extension and flexion strength. Notably, this occurred despite harvesting the hamstring tendon.

As displayed in Fig. 2a, b and Table 3, less than half of the patients had achieved an LSI > 85% for knee extension strength. Even though all patients had progressed until this stage in their rehabilitation scheme and were subsequently allowed to return-to-sport-specific training, it must be respected that the quadriceps strength retains a relevant deficit [2, 12].

From a clinical perspective, it has been shown that the effect of peripheral nerve blockage significantly reduces post-operative pain and reduces the need for opioids [1, 7]. In the light of these novel findings, we recommend using peripheral anaesthesia in all patients including athletes. The reduction in pain allows for an improved early range of motion and reduces fear of movement [18]. The reduced need for systemic application of analgesia including opioids further supports the use of regional anaesthesia since it also lowers post-operative nausea and vomiting [18, 24].

Everhart et al. described a higher re-injury risk in a cohort of FNB vs. no-block cohort which could be attributed to an extension deficit at higher speeds, since they found significant differences in LSI at 300°/sec but not at 60°/s [9]. However, they focused on extension strength only, and they did not consider the preoperative values [9]. Moreover, the flexion strength was not reported, which would have been interesting since an isolated hypoactivation of the FNB-innervated muscles should have affected the knee flexion strength less. In our data, the increase in flexion and extension torques of the non-injured leg is slightly below the injured side, making the use of the LSI as an indicator questionable. Kew et al. found that knee flexion strength is influenced by the type of anaesthesia, when they compared femoral, femoral and sciatic, Sciatic and saphenous or isolated saphenous blocks [13].

Ipsilateral H/Q-ratio seems a preferable measure to evaluate femoral nerve block compared to adductor canal block, although the presented data show no indication for differences between the groups.

The limitations of this study include its retrospective nature, which evidently was inevitable. We will strive for an RCT comparing these values. Another limitation is the fact that we cannot report patient-specific outcomes; however, it has been shown that patients’ satisfaction and perceived outcomes exhibit a strong correlation to isokinetic strength [27].

The strengths of this study are its high sample size and the well-established, objective measurement.

## Conclusions

Summarizing, this study investigated whether the type of anaesthesia (general, spinal and/or prolonged femoral nerve block) had a significant influence on the strength performance at 6 months post-operatively, which was not the case. Despite the large and homogenous sample size, there were no significant differences between the four types of anaesthesia investigated. The clinical implication of these findings is that the individual choice of anaesthesia (regional, spinal, general) remains at the patient’s and the anaesthesiologist’s discretion with no adverse effect on strength when returning to play.

## Abbreviations

ACL: Anterior cruciate ligament
GA: General anaesthesia
GA + *pFNB*: General anaesthesia with prolonged on-demand femoral nerve block
Spinal + *pFNB*: Spinal anaesthesia with prolonged on-demand femoral nerve block
Nm: Newtonmeter
SD: Standard deviation
